# Health-Promoting Lifestyles and Depression in Urban Elderly Chinese

**DOI:** 10.1371/journal.pone.0117998

**Published:** 2015-03-17

**Authors:** Yan Hua, Bo Wang, Gwenyth R. Wallen, Pei Shao, Chunping Ni, Qianzhen Hua

**Affiliations:** 1 School of Nursing, Fourth Military Medical University, Xi’an, Shaanxi Province 710032, PR China; 2 Department of Epidemiology, School of Preventive Medicine, Fourth Military Medical University, Xi’an, Shaanxi Province 710032, PR China; 3 National Institutes of Health, Clinical Center, Bethesda, MD 20892, United States of America; Nathan Kline Institute and New York University School of Medicine, UNITED STATES

## Abstract

**Objective:**

To explore health-promoting lifestyles, depression and provide further insight into the relationship between health-promoting lifestyles and depression in an urban community sample of elderly Chinese people.

**Methods:**

A cross-sectional descriptive and correlational study of 954 community-dwelling urban elderly Chinese (aged ≥ 60) was conducted from July to December 2010. Lifestyles and depression were assessed using the revised Chinese Version of the Health-Promoting Lifestyle Profile (HPLP-C) and the Geriatric Depression Scale (GDS), respectively.

**Results:**

In this cohort, 15.8% of elderly urban adults met the criteria for depression. Over half of the sample (62.1%) scored greater than 100 on the HPLP-C, with range of score sum from 55 to 160. There were significant correlations between self-actualization (OR = 1.167, 95%CI: 1.111–1.226), nutrition (OR = 1.118, 95%CI: 1.033–1.209), physical activity (OR = 1.111, 95%CI: 1.015–1.216) and depression among community-dwelling elderly Chinese.

**Limitations:**

This was a cross-sectional study. The significant associations found do not represent directional causation. Further longitudinal follow-up is recommended to investigate the specific causal relationship between lifestyles and depression.

**Conclusions:**

Depression was common with medium to high levels of health-promoting lifestyles among urban elderly Chinese people. Lifestyle behaviors such as self-actualization, good nutrition habits and frequent physical activity were correlated to fewer depressive symptoms. Healthy lifestyles should be further developed in this population and measures should be taken for improving their depression.

## Introduction

Depression is the most common psychiatric disease found in elderly people [1]. Longitudinal studies generally confirm a rising prevalence of depression [2]. Depression represents a major international public health problem associated with lower quality-adjusted life years[3], adverse health outcomes[4], all-cause mortality [5], death from suicide[6], medical illness [7] and high burden of disease[8].

Interest in elderly depression has gained increased momentum in recent years [1]. Due to methodological differences, studies on aging Asian populations reported a wide range of depression prevalence rates spanning 16.0%–29.4% [9]. As in most other countries, the proportion of elderly people is increasing every year in China. Reportedly, the percentage of Chinese elderly people over the age of 60 was 13.3% of the total population in November 1^st^, 2010[10]. In such aging populations as Mainland China, the prevalence rate of depression/depressive symptoms has been shown to vary between 3.86% and 30.8% [11–15]. The physiologic and psychosocial issues affected by depression, such as functional impairment, decreased quality of life and increased mortality in elderly people, have imposed an immense burden on individuals, families, communities and health services in China[16]. The high prevalence rate of elderly depression in China and the strong association between depression and the concomitant problems in old age demand urgent attention from prevention and intervention efforts.

A health-promoting lifestyle is one in which self-initiated, continuous, daily activity is undertaken with the deliberate aim of increasing or promoting an individual’s health and well-being. Previous studies have shown that individuals who engaged in health-promoting lifestyles will remain healthy and functional while experiencing less burden from disease and disability [17]. In different populations such as metabolic syndrome patients and sheltered homeless women, there is evidence that increased healthy lifestyles result in less depressive symptoms [18–19]. Modern populations are increasingly overfed, malnourished, sedentary, sunlight-deficient, sleep-deprived, and socially-isolated. These changes in lifestyle contribute to poor physical health and affect the incidence and treatment of depression [2]. However, there are few health promotion programs for elderly people in developing countries. Little is known about the degree of health-promoting lifestyles and how these lifestyles eventually contribute to depression among elderly people in those countries.

Using a large sample of elderly adults (aged ≥ 60) living in urban communities, this study examines the degree of health-promoting lifestyles, the prevalence of depression and the correlation between health-promoting lifestyles and depression in urban elderly Chinese. This study will elucidate the lifestyles in which elderly Chinese engage and how these lifestyles influence mental health outcomes. The study will also assist healthcare providers in developing evidence-based health-promoting strategies in the community to facilitate healthy and active lifestyles for these elderly individuals. Ultimately, this will support these individuals in achieving their highest level of health and overall quality of life.

## Method

This study was a cross-sectional descriptive and correlational study to explore health-promoting lifestyles, depression and their relationship among the urban, community-dwelling elderly in China.

### 1. Location

Xi’an is one of the oldest historical cities in China and has a vivid history and rich culture. It was called Chang’an in ancient times and is now the capital of Shaanxi province and the political, economic and cultural center of northwest China. There are six districts in the city of Xi’an, including: Xincheng district, Beilin district, Lianhu district, Yanta district, Baqiao districts and Weiyang district. Xi’an has a geographic area of 9983km^2^ and a population of 8.37 million in 2009. Out of this total population, the percentage of elderly people (aged ≥ 60) is 14.83% (1.24/8.37).

### 2. Samples

The sample size was estimated according to the following formula N = t^2^×P×Q/d^2^ (P: Prevalence, Q = 1-P), which is commonly used in cross-sectional studies in epidemiology. Previous studies estimated that the average prevalence of depressive disorders was 17.3% in China [11–15]. If d = 0.15P and α = 0.05, the estimated sample would be 850 participants. An additional 15% was added to this sample estimate in anticipation that the final sample would include elderly adults who would not consent to participate in the survey. Thus the final sample size was estimated to be 979.

Multi-stage stratified random cluster sampling was used. First, four districts were randomly selected from the six districts of Xi'an. Second, two or three street areas were randomly selected in every selected district. Third, one community was randomly selected in every selected street area. In all, nine communities were randomly selected from July to December in 2010. All the elderly adults aged 60 years and older in the nine selected communities were invited to a face-to-face interview to answer a standardized questionnaire. Exclusion criteria included: people in long-term care, with terminal diseases, or with moderate/severe cognitive impairment and currently suffering from deafness, aphasia or other communication disorders.

### 3. Ethical issues

The study was approved by the Institutional Review Board of the Fourth Military Medical University. Each participant was informed verbally about the aims and methods of the survey before the interview. They were assured that the data would be treated confidentially. Participation was voluntary, and written consent was obtained from each participant.

### 4. Measures

The questionnaire used during the face-to-face interviews included individual demographics, the Simplified Chinese Version of the 40-item Health-Promoting Lifestyles Profile and the 30-item Geriatric Depression Scale and Personal Data Form.

#### The Simplified Chinese Version of the 40-item Health-Promoting Lifestyles Profile (HPLP-C)

The HPLP-C is used to identify patterns of health-promoting lifestyles and health-promoting behaviors which are conceptualized as a multidimensional pattern of self-initiated actions and perceptions that serve to maintain or enhance the level of wellness[20]. The HPLP-C consists of 40 items which encompass 6 subscales: nutrition, physical activity, health responsibility, interpersonal relations, self-actualization and stress management.

The HPLP-C has been widely used in different settings [19]. It was originally developed by Walker, Sechrist and Pender (*1987*) and later revised as the HPLP-II [21]. The revised version contained 52 items, and has been widely used [19, 22].In 1997, it was translated into a Chinese (Taiwanese) version, and both reliability and validity of the translation were verified [23]. Finally, a 40-item Chinese (Taiwanese) version of HPLP, namely the HPLP-C, was constructed with six subscales [23–24]. Reliability and validity for the HPLP-C were found to be excellent for Chinese elderly people in our previous study [25]. Internal consistency reliability was reported as 0.91 for the total scale and a ranged from 0.67 to 0.88 for the six subscales [25].

Items are measured using a four-point Likert scale (1 = never, 2 = sometimes, 3 = often and 4 = routinely), with higher scores indicating healthier lifestyle choices. Individual responses are score as a total score as well as six subscale scores. The higher the total score, the healthier the lifestyle is. The total score ranges from 40 to 160and is divided into four grades (40–69, 70–99, 100–129 and 130–160 indicating poor, general, good and excellent lifestyle, respectively). According to the total score, lifestyle can be divided into healthy and unhealthy categories (the total score of 40–99 and 100–160 indicates healthy and unhealthy lifestyle, respectively). The average score for each subscale is also calculated to reflect the frequency of specific lifestyle activities. Higher average subscale scores indicate higher engagement in the lifestyle activity.

#### The Geriatric Depression Scale (GDS)

The GDS is a 30-item scale developed to assess depressive symptoms specifically for use in elderly populations [26]. The GDS has been widely used in different settings [27–29], including community studies [30]. The scale possesses adequate sensitivity (92%) and specificity (89%) [31–32]. In a previous study of Chinese elderly aged 60 or older, the GDS was validated in a psychiatric outpatient sample [33]. Its sensitivity was 70.6%, with a specificity of 70.1%. Internal consistency reliability was 0.89 and the test-retest reliability was 0.85. Maximum overall score of GDS is 30. Participants with a score of 11 points or higher were considered to have positive depressive symptoms. According to the continuous GDS score, the degree of depressive symptom is defined: mild depression as a GDS score between 11 and 20, and moderate/severe depression as a GDS score greater than 20. For this study ‘depression’ was operationally defined as the presence of positive depressive symptoms according to the GDS.

#### The Personal Data Form

This form was developed by the researcher to obtain data related to demographics and some individual characteristics such as age, gender, education, monthly income, current marital status, physical condition and body mass index (BMI, derived from height and weight measurements).

### 5. Statistical analysis

All completed questionnaires were anonymized with individual numbered. Double data entry was conducted by two independent professional data-entry workers using Epidata Version 3.1. Computer and manual checks ensured accurate data coding. Statistical analyses were performed using the software package SPSS 17.0. Descriptive statistics including frequencies, percentages, means and standard deviations (SD) were used to examine sample characteristics. Besides descriptive statistics, analysis of variance (ANOVA) was used to explore the differences in depressive symptoms based on specific sample characteristics.

To estimate the correlation between health-promoting lifestyles and depression, multivariate logistic regression analysis was performed, with or without significant depression as the dependent variable, and 6 dimensions of HPLP as independent variables. Coefficient values, odds ratios (OR) and 95% confidence intervals (CI) were used to quantify the strength of the correlations. The statistical significance for all tests was set at *P* < 0.05.

## Results

### 1. Sample characteristics

A total 979 elderly adults from nine selected communities in Xi'an were invited to participate in the study, and 954 completed the questionnaire. Twenty-five elderly adults were not able to complete the questionnaire. Thus the response rate for completed questionnaires was 97.4% (954/979).

Of the 954 participants, there were 350 (36.7%) males and 604 (63.3%) females. Ages of respondents ranged from 60 to 93 years, with a mean age of 70.88 years (SD = 7.23). Only 10.7 percent of the sample had post secondary education. The socio-demographic characteristics of the participants are presented in Table 1.

**Table 1:** Demographic characteristics of the sample (n = 954).

Variable	N	Percentage
**Age (years)**		
60–69	420	44.0
70–79	419	43.9
80 and above	115	12.1
**Gender**		
Male	350	36.7
Female	604	63.3
**Education**		
No formal education	192	20.1
Primary	231	24.2
Junior high school	225	23.6
Senior high school/technical secondary school	204	21.4
Post-secondary and above	102	10.7
**Current marital status**		
Married	679	71.2
Single/divorced/widowed	275	28.8
**Monthly income (RMB)**		
Less 1000	261	27.4
1000–1999	532	55.8
2000 and above	161	16.9
**BMI (kg/m^2^)**		
<18.5	52	5.5
18.5–23.9	405	42.5
24.0–27.9	299	31.3
≥28	198	20.8

### 2. Health-promoting lifestyle in elderly people

The total score of HPLP-C varied from 55 to 160. The total score of 62.1% participants was more than 100, with a mean score of 104.94 and standard deviation of 17.40. The actual total scores for each of the subscales were as follows: nutrition (16.44); health responsibility (15.23); self-actualization (23.29); interpersonal relations (17.60); sports activity (8.00); and stress management (24.39). Considering the difference of the number of items among six subscales, the average score of each subscale was calculated to compare the lifestyle behaviors among six subscales. The average score and range of actual total score are shown in Table 2.

**Table 2:** Score of HPLP-C subscales (n = 954).

Variable	Standard Total Score	Range of Actual Total Score (min-max)	Actual Total Score	Average Score (Mean±SD)
Nutrition	20	5–20	16.44	0.82±2.47
Interpersonal relations	24	6–24	17.60	0.73±4.17
Self- actualization	32	8–32	23.29	0.73±5.03
Stress management	36	9–36	24.39	0.68±5.22
Physical activity	16	4–16	8.00	0.50±2.72
Health responsibility	32	8–32	15.23	0.48±4.24
Total Score	160	55–160	104.94	104.94±17.40

### 3. Depression in elderly people

The score of GDS ranged from 0 to 29, with a mean score of 5.82 (SD = 5.42). Out of 954 participants, 151 (15.8%) had positive depression, in which 123 (12.9%) met the criteria of mild depression and 28 (2.9%) were moderately/severely depressed. In Table 3, participants’ characteristics by depression are presented. Low monthly income was associated with greater probability of depression(*P*<0.05).

**Table 3:** Participants’ characteristics by depression status (n = 954).

Variable	Depression		Non-depression		χ^2^	*P*
	n	%	n	%		
**Age (years)**						
60–69	64	6.7	356	37.3	2.501	0.286
70–79	63	6.6	356	37.3		
80 and above	24	2.5	91	9.5		
**Gender**						
Male	45	4.7	305	32.0	3.662	0.056
Female	106	11.1	498	52.2		
**Education**						
No formal education	39	4.1	153	16.0	7.535	0.110
Primary school	40	4.2	191	20.0		
Junior high school	32	3.4	193	20.2		
Senior high school/technical secondary school	31	3.2	173	18.1		
Post-secondary school and above	9	0.9	93	9.7		
**Current marital status**						
Married	98	10.3	581	60.9	3.441	0.064
Single/divorced/widowed	53	5.6	222	23.3		
**Monthly income (RMB)** *						
Less 1000	70	7.3	191	20.0	33.993	0.000
1000–1999	67	7.0	465	48.7		
2000 and above	14	1.5	147	15.4		
**BMI (kg/m2)**						
<18.5	12	1.3	40	4.2	4.123	0.249
18.5–23.9	70	7.3	335	35.1		
24.0–27.9	41	4.3	258	27.0		
≥28	28	2.9	170	17.8		

* Renminbi (RMB).

### 4. Correlation between the lifestyle and depression

Table 4 shows the correlation between lifestyles and depression among elderly adults. Multivariate Logistic Regression Analysis suggests there was significant correlation between self-actualization (OR = 1.167, 95%CI: 1.111–1.226), nutrition (OR = 1.118, 95%CI: 1.033–1.209), physical activity (OR = 1.111, 95%CI: 1.015–1.216) and depression among elderly people (*P*<0.05).

**Table 4:** Logistic regression analysis of lifestyles and depression.

	Regression coefficient	Standard error	χ^2^	*P*	OR	95%CI
Self- actualization	0.155	0.025	38.062	0.000	1.167	1.111–1.226
Nutrition	0.111	0.040	7.715	0.005	1.118	1.033–1.209
physical activity	0.105	0.046	5.231	0.022	1.111	1.015–1.216
Interpersonal relations	0.028	0.029	0.922	0.337	1.029	0.971–1.089
Stress management	−0.030	0.025	1.447	0.229	0.970	0.923–1.019
Health responsibility	−0.010	0.029	0.114	0.735	0.990	0.935–1.049

## Discussion

### 1. Health-promoting lifestyle in elderly Chinese

Elderly adults in China were found to participate in a variety of health-promoting behaviors, which indicated both their ability and interest in their personal health and wellness. Health-promoting lifestyles are a series of behaviors which guide an individual’s family, community and society to improve peacefulness, happiness and realize health proficiency. That is to say, these health-promoting behaviors involve any activities that focus on attaining higher levels of health, peacefulness and happiness [20]. In our study, 62.1% of elderly adults were found to engage in healthy lifestyles. The level of health-promoting lifestyles in this elderly population is similar to those found in other community-dwelling adults [34–35]. These results indicate most of the elderly adults sampled would like to engage in positive daily lifestyles to promote sustained levels of optimal health as well as overall quality of life. The current study also showed elderly adults scored highest on the nutrition subscale, lowest on the physical activity subscale and health responsibility subscales among the six subscales. These results are similar to others from diverse population groups such as Thailand physical therapy students[36] and Chinese community-dwelling adults[37]. The findings suggest that interventions to improve knowledge of health responsibility and sports activities that promote health should be an area of focus among community-dwelling elderly Chinese. Some behaviors such as help-seeking for health maintenance and improvement are based on health responsibility. It is only through the realization of the importance of health responsibility and health-promoting lifestyles that these elderly individuals can actively engage in measures to promote a sustained positive daily lifestyle that includes increased physical activity.

### 2. Depression in elderly Chinese

The current study found depression was common among community-dwelling elderly Chinese. Out of the participants, 15.8% met the criteria of depression, which was much lower than that of the Mexico study (21.7%) and some studies in Mainland China (25.5%–30.8%) [12–14]. Due to the varying methodologies, such as the different samples, sampling procedures, data collection and diagnostic criteria used, any direct comparisons are limited. Furthermore, the differences in social and economic development between study locations may lead to different results. Xi’an is an old city in western China and its development is not as advanced as the cities in eastern and southern China. As a result, the pace of people’s life in Xi’an is lower than those in the eastern and southern cities of mainland China. We suspect these differences may represent a factor that influenced both health-promoting lifestyles and the depression rate. Notably, compared to the prevalence (27.0%) found in our previous 2008 study [15], the rate of depression in elderly people decreased. The result is inconsistent with Brandon’s report [2]; however, the finding is desirable. In recent years, the increase in the standard of living among elderly people and the improvement in mental health literacy in China may contribute to this decreasing prevalence of depression.

Univariate analysis revealed significant associations between income levels and depression. The participants with a maximum income of 2000 and above Renminbi (RMB) had less risk of depressive disorders compared to those with maximum incomes of 2000 or less RMB, which was consistent with Wong’s research [38]. One previous study showed there was no significant correlation between age and depressive disorders [30], which was confirmed in our current study. Previous studies report a significant effect of the number of years of education and current marital status on the risk of depression [2, 38–39]. Our findings were inconsistent with these results, there was no significant relationships between education; attainment, marital status and depression in our study. To some extent, the differences among samples may be one of the major reasons for these conflicting results. Further study should be conducted to verify these findings.

### 3. Correlation between lifestyle and depression

Consistent with previous studies [18], the current study found health-promoting lifestyles and depression were negatively correlated. Among the six subscales of HPLP, self-actualization, nutrition and physical activity had significantly negative correlations with depression in urban elderly Chinese. Desirable changes in lifestyles among elderly adults such as self-actualization (OR = 1.167, 95%CI: 1.111–1.226), good nutritional habits (OR = 1.118, 95%CI: 1.033–1.209) and frequent physical activity (OR = 1.111, 95%CI: 1.015–1.216) were independent predictive factors for lower depression prevalence. The relationship between self-actualization, physical activity and depression has been proved in previous studies [18, 40]. Recently, a systematic review of cohort studies on diet and the risk of unipolar depression in adults reported diet may influence the risk of depression. Variables inversely associated with depression risk were the consumption of nutrients such as folate, omega-3 fatty acids and monounsaturated fatty acids; foods such as olive oil and fish; and a diet rich in fruits, vegetables, nuts and legumes[41]. Our study found there was a negative correlation between nutrition and depression among elderly adults, which suggested that strengthening healthy-eating patterns may reduce the risk of depression among elderly adults. However, the specific association between nutrients and risk of depression still needs to be studied further through longitudinal, prospective cohort studies in the future.

### 4. Study limitations and strengths

Some limitations of this study should be considered. It was a cross-sectional study performed in Xi’an, a city in western China where regional and environmental factors may influence the lifestyles of elderly people. In addition, the design of our study was a cross-sectional, multistage community survey. The significant associations found do not suggest directional causation. Further research, using a longitudinal design, is recommended in order to investigate the detailed causal relationship between lifestyle behaviors and depression.

This is one of the largest studies to explore lifestyles and depression among Chinese elderly people. It is a multi-centre study among community-dwelling Chinese elderly people, which provides valuable and previously unavailable information regarding the correlation between lifestyle and depression among elderly Chinese. The study will be very important for guiding early interventions to promote healthy lifestyles and potentially decrease depression among elderly people.

## Conclusions

The population of China is aging. More emphasis should be put on improving quality of life outcomes among elderly people. Our study provides useful information on health promoting lifestyles and the relationships between lifestyles and depression among elderly adults in a developing country. In our study, 15.8% of elderly adults reported depressive symptoms. According to HPLP-C estimates, health-promoting lifestyles of elderly Chinese in this sample were at the medium level and above. Lifestyle behaviors such as self-actualization, good nutritional habits and frequent physical activity are correlated to fewer depression disorders among community-dwelling elderly adults in China. In developing countries, the benefits of promoting sustained health behavior and lifestyle changes are crucial for elderly adults. To promote healthy lifestyles, it is needed to improve their depression.
